# Skeletal Muscle Fatigability in Heart Failure

**DOI:** 10.3389/fphys.2019.00129

**Published:** 2019-02-21

**Authors:** Manda L. Keller-Ross, Mia Larson, Bruce D Johnson

**Affiliations:** ^1^Divisions of Physical Therapy and Rehabilitation Science, Department of Rehabilitation Medicine, Medical School, University of Minnesota, Minneapolis, MN, United States; ^2^Department of Cardiovascular Diseases, Mayo Clinic and Foundation, Rochester, MN, United States

**Keywords:** heart failure, fatigue, fatigability, exercise intolerance, skeletal muscle

## Abstract

Evidence suggests that heart failure (HF) patients experience skeletal muscle fatigability in the lower extremity during single-limb tasks. The contribution of skeletal muscle fatigability to symptoms of exercise intolerance (perceived fatigue and dyspnea) is relatively unclear. Symptomatic or ‘perceived’ fatigue is defined by the sensations of exhaustion or tiredness that patients experience either at rest or while performing a motor task. Although factors that contribute to symptoms of fatigue in patients with HF are multifactorial; the skeletal muscle likely plays a major role. Skeletal muscle fatigability, as opposed to symptomatic fatigue, is an objective measure of a reduction in muscle force or power or reduced ability of the muscles to perform over time. Indeed, evidence suggests that patients with HF experience greater skeletal muscle fatigability which may contribute to a diminution in motor performance and the overall symptomatology that is hallmark of exercise intolerance in HF. This review will discuss (1) skeletal muscle fatigability in patients with HF, (2) the mechanisms contributing to locomotor skeletal muscle fatigability in HF and (3) the relationship of fatigability to symptoms of perceived fatigue and exercise intolerance in HF patients. Evidence suggests that cardiac dysfunction alone does not contribute to exercise intolerance. Therefore, mechanisms of skeletal muscle fatigability and their contribution to symptoms of fatigue and exercise intolerance, is an increasingly important consideration as we develop rehabilitative strategies for improving motor performance and functional capacity in patients with HF.

## Introduction

Heart failure (HF) is the inability of the heart to supply the periphery with adequate nutrients and oxygen. HF is either defined by the inability of the heart to pump blood adequately (HF with reduced ejection fraction, HFrEF) or the inability of the heart to fill adequately (HF with preserved ejection fraction, HFpEF) (Hunt et al., 2009). Although there are many causes of HF, the end result is a systemic illness that affects multiple organ systems, including skeletal muscle (Wilson, 1995). Exercise intolerance, low exercise capacity accompanied by symptoms of dyspnea and fatigue, is a hallmark of HF (Wilson and Ferraro, 1983; Wilson et al., 1984b; Piepoli et al., 1995; Wilson, 1995). Mechanisms of exercise intolerance in patients with HF are multi-factorial with several excellent reviews detailing the importance of skeletal muscle to exercise intolerance (Lipkin and Poole-Wilson, 1986; Wilson and Mancini, 1993; Poole et al., 2012, 2018; Hirai et al., 2015). Symptoms of exercise intolerance are commonly dissociated with measurements of resting cardiac output, ejection fraction and left atrial pressure in HF (Franciosa et al., 1979; Higginbotham et al., 1983; Szlachcic et al., 1985; Lipkin and Poole-Wilson, 1986). However, the contribution of skeletal muscle fatigability and associated mechanisms to perceptions of fatigue and exercise intolerance are unknown and will be discussed in this review. Indeed, the purpose of this review is to provide an overview of skeletal muscle fatigability and discuss the extent to which fatigability contributes to symptoms of fatigue and exercise intolerance in patients with HF. Although both HFrEF and HFpEF likely exhibit skeletal muscle fatigability, the majority of the work in fatigability has been conducted in HFrEF and will therefore be the emphasis of this review. Further, the majority of the fatigability work in HF was performed in the 1980s and 1990s. These studies will be the focus of the discussion, with more recent work in muscle to delineate potential mechanisms of fatigability. In final, this review will highlight the critical need for further characterization and delineation of mechanisms of skeletal muscle fatigability in HF.

Symptomatic or ‘perceived’ fatigue is defined by the sensations of weariness, increasing sense of effort, mismatch between effort expended and actual performance or exhaustion (Kluger et al., 2013). Fatigue is measured subjectively, often times by a rating of perceived exertion scale (Borg, 1974; Borg et al., 1987) or merely by the presence of its existence in clinical populations. A clear distinction, however, needs to be made between perceived fatigue as described by patients and skeletal muscle fatigability (Kluger et al., 2013).

Fatigability, commonly called performance fatigability, is an objective measure of a *reversible*, reduction in muscle force or power or the reduced ability of the muscles to perform over time (Gandevia, 2001). Mechanisms of fatigability can occur upstream of the neuromuscular junction (central or neural mechanisms) and/or at or below the neuromuscular junction (peripheral mechanisms). Central mechanisms can include motivation, inhibition at the motor cortex or a reduction in drive to the motor neurons or inability to fully recruit motor units (Gandevia, 2001). Peripheral mechanisms include impairment at the neuromuscular junction, changes in blood flow, metabolism, contractile properties and calcium kinetics (Fitts, 1994, 2008; Kent-Braun et al., 2012). Mechanisms of greater fatigability in patients with HF are not clear, but clinically relevant, and likely to contribute to perceptions of fatigue and exercise intolerance. Although there is no consensus, there is evidence to suggest, that mechanisms of fatigability are likely a combination of deconditioning and the pathophysiology of HF (Buller et al., 1991; Piepoli et al., 1995; Piepoli and Coats, 2013).

## Are Patients With HF More Fatigable?

**FIGURE 1:**
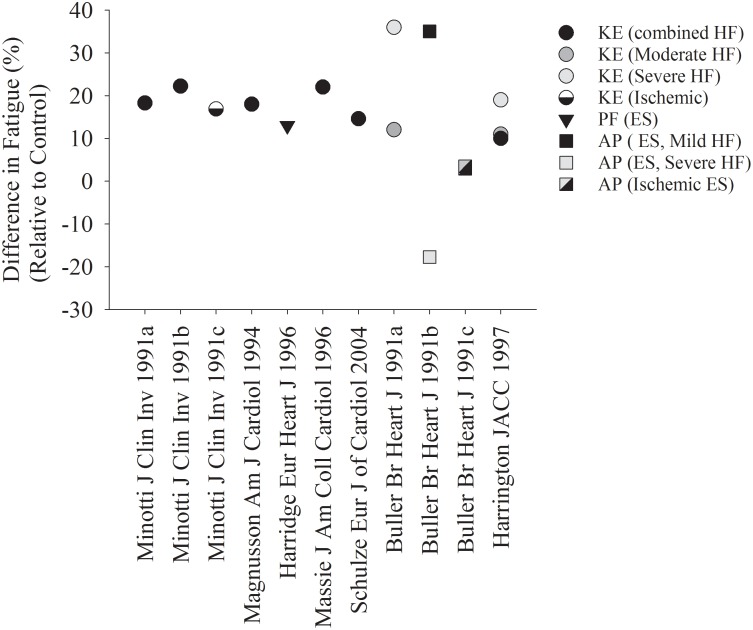
Reduction in Strength after a fatiguing contraction in HF, relative to controls. Each symbol represents the reduction in strength after various fatiguing interventions, normalized to the control group in each respective study. The majority of studies demonstrate a greater fatigability in HF patients compared with controls. When studies used multiple protocols (Buller et al., 1991; Minotti et al., 1991), each protocol was indicated in its own column. CTL, control; HF, heart failure; KE, knee extensors; PF, plantar flexors; AP, adductor pollicis; ES, electrical stimulation.

**FIGURE 2:**
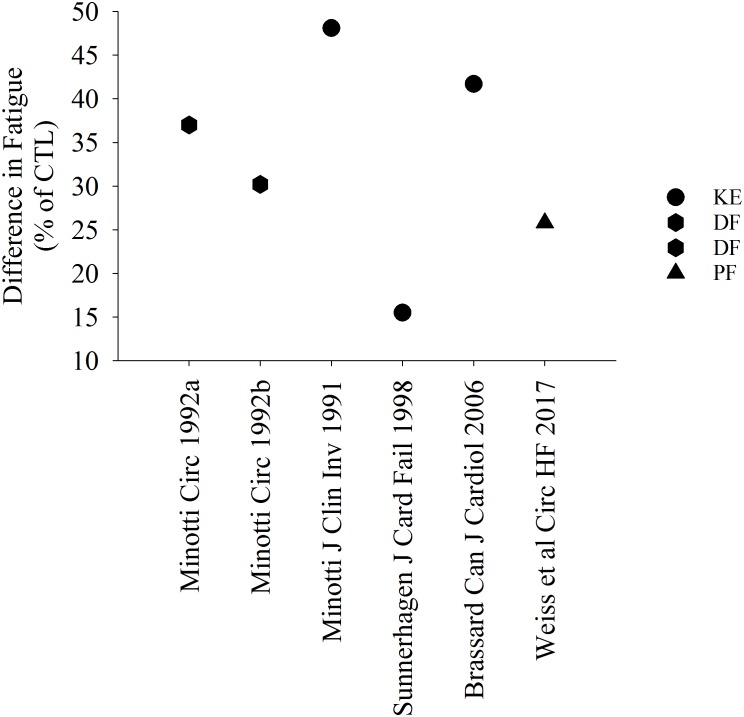
Percent difference in time to task failure in patients with HF. Data in HF is represented as a relative value in comparison with controls. All studies demonstrated a briefer time to failure or greater fatigability in HF. DF, dorsiflexor muscles.

Several studies suggest that patients with HF are more fatigable when compared with controls (Figure 1, 2). Figure 1 represents studies that measured the reduction in strength after various single-limb fatiguing interventions. Figure 2 represents the studies that measured time to task failure or how long participants could hold a submaximal or maximal contraction, or in one case of isometric intermittent contractions, how many contractions the participants were able to perform (Minotti et al., 1992b). In both Figure 1, 2 the reduction in strength or time to failure for the HF participants was normalized to the CTL participants. For brevity, if studies sorted out differences in fatigability based on severity of HF according to the New York Heart Association (NYHA) classification scale (Weiss et al., 2017), this is discussed in the text but averaged for Figure 2. Fatigability and mechanisms that contribute to fatigability are known to be task dependent (Hunter, 2018) and may also be dependent on the muscle (Hunter et al., 2004; Senefeld et al., 2017). This means that fatigability for isometric (static or intermittent), dynamic tasks, maximal or submaximal may alter the performance of the muscle and the mechanisms that cause the muscle to fatigue are dependent on that task and may be different for each muscle. Although the literature in HF is sparse compared with healthy adults or aging literature, the majority of the studies demonstrate that patients with HF are considered more fatigable in that they experience a greater reduction in strength as well as a briefer time to failure when compared with control participants. Further, for the knee extensor and plantar flexor muscles, fatigability was greatest for patients with the most severe HF symptoms (Buller et al., 1991; Harrington et al., 1997; Weiss et al., 2017). This was not consistent, however, for the adductor pollicis muscle (Buller et al., 1991). Buller et al. (1991) observed that when the adductor pollicis was electrically stimulated, HF patients who identified as mild HF were more fatigable compared with controls. Surprisingly, however, patients that identified as severe HF were less fatigable then control participants (Buller et al., 1991 in Figure 1). Further, when blood flow was occluded to the adductor pollicis muscle during electrical stimulation, there was little difference between both mild and severe HF patients and control participants (Buller et al., 1991). The difference between lower and upper extremity fatigability in patients with HF is not completely understood but may be related to a greater deconditioning of the lower extremity muscles in patients with HF. However, this should be taken with caution as very few studies have measured skeletal muscle fatigability of the upper extremity in patients with HF and these studies have primarily used electrical stimulation vs. volitional fatiguing contractions (Buller et al., 1991).

## Mechanisms of Skeletal Muscle Fatigability

### Influence of Muscle Mass and Strength to Skeletal Muscle Fatigability

**FIGURE 3:**
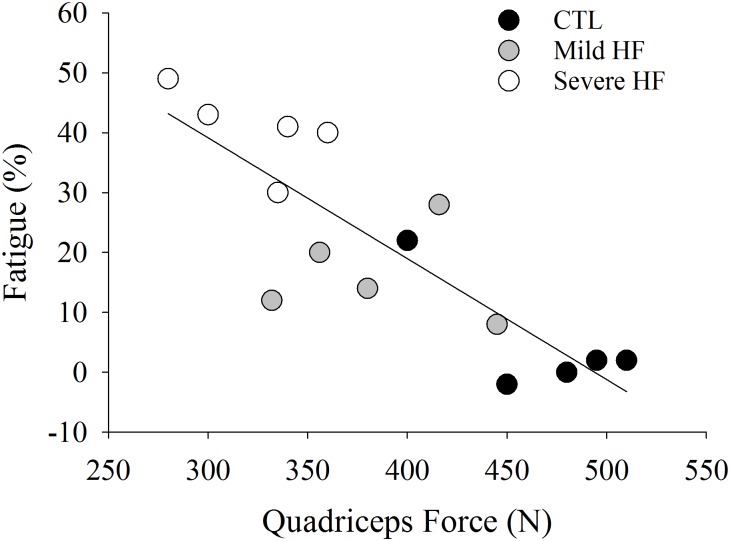
Data extracted from Buller et al. (1991). Quadriceps force was strongly associated with fatigue (%) (*r* = 0.81, *P* < 0.001). Individuals who were weaker (severe HF patients) had the greatest fatigability (%).

Skeletal muscle strength plays a major role in fatigability in healthy adults (Hunter and Enoka, 2001; Keller-Ross et al., 2014). During isometric tasks, individuals that have weaker muscles generally demonstrate a longer time to task failure or greater resistance to fatigue, which could be due to greater blood perfusion during the task and/or a greater proportion of type I (oxidative) muscle fibers (Hunter, 2009). Whether or not skeletal muscle strength is affected in patients with HF is equivocal in the literature. Some studies suggest that skeletal muscle strength is relatively preserved in patients with HF (Minotti et al., 1992a; Harridge et al., 1996; Massie et al., 1996; Brassard et al., 2006), but others indicate strength is reduced (Buller et al., 1991; Magnusson et al., 1994; Sunnerhagen et al., 1998; Schulze et al., 2004). Of significance and similar to what we observe in the relationship between HF and fatigability, these latter studies observed that patients with the most severe HF exhibit the greatest deficits in strength (Buller et al., 1991). Indeed, patients with severe HF, also had the weakest knee extensor muscles and greater fatigability after intermittent isometric contractions at 40% of maximal voluntary contraction for 20 min (*r* = 0.81, *p* ≤ 0.01, Figure 3). This is in contrast to what is observed in healthy adults when time to task failure is measured during isometric fatiguing contractions (Hunter, 2009). In the case of Buller et al. (1991), the stronger individuals (less severe HF and control participants) had greater strength and were less fatigable (less of a reduction in strength) than weaker individuals who also identified as having severe HF symptoms. To further illustrate the importance of strength to fatigability, patients with severe HF that exhibit cachexia (loss of muscle mass and body weight) have reduced skeletal muscle strength and experience greater reductions in maximal strength (21.1% ± 1.9) vs. non-cachectic HF patients (13.9 ± 1.6%) after a fatiguing intervention (Anker et al., 1997b).

**FIGURE 4:**
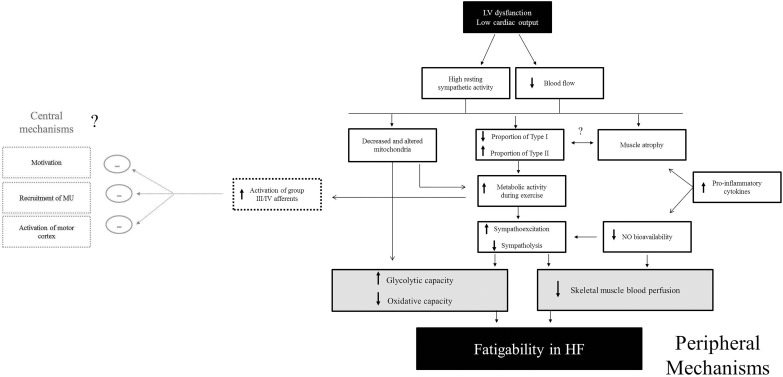
Proposed mechanisms of fatigability in HF patients. Mechanisms contributing to muscle fatigability in HF are likely a cascade of events that may be initiated by high sympathetic activity and reduced cardiac output. This review indicates that peripheral factors likely play a large role in fatigability. In particular, skeletal muscle perfusion and altered muscle metabolism appear to be observed as major contributing factors (shown in gray). Although central factors have not yet been shown to contribute to fatigability in HF, more research needs to be conducted to directly measure neural mechanisms of fatigability in these patients. Because greater activation of skeletal muscle (group III/IV) afferents are known to inhibit motor activity (Garland and Kaufman, 1995), the possibility of greater inhibition of the central nervous system by exaggerated activity of group III/IV afferent (outlined in gray boxes) exists.

Mechanisms contributing to a reduction in strength in patients with HF are likely linked to skeletal muscle atrophy. Several studies demonstrate a strong relationship between a reduction in strength and a loss of muscle mass (Buller et al., 1991; Minotti et al., 1993; Magnusson et al., 1994). The causes of muscle atrophy in patients with HF are likely multifactorial and include disuse, reduced blood flow, neural and hormonal factors and/or intrinsic skeletal muscle alterations (Wilson et al., 1984b; Mancini et al., 1992; Gosker et al., 2000; Miller et al., 2009, 2010). For example, metabolic abnormalities in HF affect various endocrine systems leading to an imbalance of catabolic and anabolic function which results in progressive catabolic state in advanced stages of disease (Mancini et al., 1992). The neurohumoral activation is accompanied by increased serum levels of pro-inflammatory cytokines (TNFα, IL-1β and IL-6) (Torre-Amione et al., 1996). Several studies indicate that systemic markers of pro-inflammatory cytokines contribute to muscle atrophy in patients with HF (Anker et al., 1997a; Hambrecht et al., 2002). Collectively, these studies suggest that maintaining skeletal muscle mass and strength may be important for fatigue resistance in patients with HF. It remains to be determined, however, if and how reduced strength or muscle atrophy causes fatigability or if it is a coincidental and/or simultaneous occurrence with the change in fiber type composition (Figure 4) (Minotti et al., 1993).

### Contribution of Neural Mechanisms to Skeletal Muscle Fatigability in HF

Central (neural) mechanisms of skeletal muscle fatigability is defined by a reduction in voluntary activation from contributions upstream of the neuromuscular junction (Gandevia, 2001). There are limited studies that have assessed neural mechanisms of fatigability in patients with HF, however, these studies suggest that neural mechanisms are likely not a major cause of fatigability. For example, Harridge et al. (1996) demonstrate a 30% greater fatigability in patients with HF, but this could not be attributed to poor neural drive. Minotti et al. (1992b), using a dynamic and sustained isometric contractions of the dorsiflexors, reported that neural drive was a limiting factor to force production during fatiguing exercise, but this limitation was similar in patients with HF and control participants (Minotti et al., 1992b). Further, a more recent study demonstrated that patients with HF had a similar reduction in skeletal muscle strength and voluntary activation (measured via transcranial magnetic stimulation) of the quadriceps muscle after a peak exercise test (Hopkinson et al., 2013). Alternatively, greater skeletal muscle fatigability was correlated with a reduction in electromyographic (EMG) activity of the knee extensor muscles during a maximal isometric fatiguing contraction (Schulze et al., 2004). The authors recognize this finding as a pronounced decrease in neuromuscular activity in HF, however, surface EMG does not only represent neural activation, but also represents changes distal to the neuromuscular junction (Farina et al., 2004). Collectively, although neural fatigue appears to contribute to skeletal muscle fatigability in HF, this does not appear to be different from those without HF and is not a major contribution to the greater fatigability observed in patients with HF.

### Contribution of Peripheral Mechanisms to Skeletal Muscle Fatigability in HF

Mechanisms contributing to fatigability that occur at or distal to the neuromuscular junction (NMJ) are defined as peripheral. This includes; impairment of the NMJ to transmit the action potential along the sarcolemma and t-tubule, alterations to the contractile properties, muscle metabolism and blood flow and intrinsic changes to the skeletal muscle. The NMJ can be assessed by the compound muscle action potential or the M wave, which is the EMG response to maximal stimulation to the motor nerve or muscle. The M wave was found to be similar in HF and CTL participants during an intermittent isometric fatiguing contraction, suggesting that the integrity of the NMJ is well-preserved with fatigue in HF (Minotti et al., 1992b). As such, the majority of the evidence suggests that alterations peripheral of the NMJ are likely the cause of greater fatigability in HF.

#### Altered Contractile Properties and Muscle Metabolism in HF

Several studies suggest a loss in proportion of type I and type IIa fibers, a gain in type IIx fibers (Mancini et al., 1989; Sullivan et al., 1990; Harridge et al., 1996; Sunnerhagen et al., 1998) and a lower level of oxidative enzymes among HF patients than in controls (Sullivan et al., 1990; Drexler et al., 1992; Massie et al., 1996; Sunnerhagen et al., 1998). For example, a faster time to peak tension in the plantar flexor muscles and a faster ½ relaxation time in the knee extensor muscles was observed in HF with 54% and 45% greater fatigability in HF patients, respectively (Harridge et al., 1996). The faster ½ relaxation time in patients with HF suggests faster calcium kinetics and specifically calcium reuptake in the sarcolemma (Inghilleri et al., 1993). Further, Brassard et al. (2006) demonstrated that a 39% briefer endurance time in patients with HF was correlated with lower oxidative enzymes [citrate synthase (CS), 3-hydroxyacyl-CoA dehydrogenase (HADH)] and greater glycolytic enzymes [phosphofructokinase (PFK)]. This suggests that the greater fatigability in the vastus lateralis in patients with HF was likely due to lower oxidative capacity. These patients also had an elevated median frequency of surface EMG suggesting a greater reliance on type II fiber recruitment (Brassard et al., 2006). These findings confirmed results of greater oxidative capacity and lower glycolytic capacity found in earlier studies (Sullivan et al., 1990, 1991). As such, lactate production was found to be greater during submaximal exercise in patients with HF (Sullivan et al., 1990, 1991). Several studies that use phosphorus nuclear magnetic resonance (^31^P NMR) demonstrate altered metabolism, such that a greater increase in the ratio of inorganic phosphate to phosphocreatine (Pi/PCr) was found during the higher intensity workloads in patients with HF (Wiener et al., 1986). Consistent with these findings, evidence suggests that HF patients demonstrate a reduced number of mitochondria and the mitochondria to be structurally altered in skeletal muscle of HF (Drexler et al., 1992; Hambrecht et al., 1995). This is likely due to the elevated sympathetic activity and release of neurohumoral factors, such as catecholamines or renin-angiotensin-aldosterone system (Palaniyandi et al., 2010). In summary, several lines of evidence suggest that HF muscles demonstrate a higher glycolytic and lower oxidative capacity during exercise which contributes to skeletal muscle fatigability in HF (Figure 4).

Critical power represents the highest *power* output sustained without loss of homeostasis (Jones et al., 2010) and subsequently the highest rate of oxidative metabolism that can be sustained without a progressively increasing contribution to energy turnover from substrate-level phosphorylation (anaerobic glycolysis) (Jones et al., 2010). While challenging to assess, it has been suggested that critical power is reduced in patients with HF, which likely contributes to a greater skeletal muscle fatigability (Jones et al., 2010) and therefore reduced tolerance to exercise (Poole et al., 2012).

#### Altered Blood Flow in Patients With HF

Several aspects of muscle blood flow control are disrupted in HF. A decrease in skeletal muscle perfusion during exercise is a powerful stimulus for early anaerobic metabolism (Pernow et al., 1975; Walker et al., 1982) which has been demonstrated in HF (Zelis et al., 1974; Wilson et al., 1984a,b). Part of the blood flow discrepancy in HF may also be due to an intrinsic abnormality in limb vasodilatory capacity (Zelis et al., 1968), greater sympathetic activity (Notarius et al., 2001; Osman and Lee, 2015) greater impedance of the muscle pump from post capillary resistance (Zelis et al., 1974; McAllister et al., 1993; Shiotani et al., 2002); however, muscle fiber capillary density appears to be normal (Lipkin et al., 1988). Nitric oxide (NO) bioavailability is also compromised, which would constrain sympatholysis (the ability to oppose α-adrenergic vasoconstriction and shear stress-mediated vasodilation) (Thomas et al., 2001). Importantly, greater increases in pro-inflammatory cytokines such as TNF-α and IL-1β levels may also impact NO bioavailability (Batista et al., 2009) leading to greater vasoconstriction of blood flow to skeletal muscle (Figure 4).

Although no studies have investigated leg blood flow during fatiguing contractions, leg blood flow was shown to be impaired during knee extensor exercise at various workloads (Amann et al., 2014). This was likely due to greater skeletal muscle group III/IV afferent feedback, causing greater sympathoexcitation and reduced vasodilatory capacity at the site of the muscle (Amann et al., 2014). Other studies using maximal whole body exercise have also demonstrated impaired blood flow (Magnusson et al., 1997; Wilson et al., 1983, 1984a,b) which could in part, be due to competition of blood flow between locomotor and respiratory muscles (Olson et al., 2010). Measurement of blood flow during a smaller muscle exercise, such as with the forearm muscles suggests that blood flow is impaired in some studies (Zelis et al., 1974; Longhurst et al., 1976), but not others (Wiener et al., 1986; Magnusson et al., 1997). Differences in cardiac dysfunction and severity of HF likely account for the difference in findings.

Oxygen uptake (VO_2_) kinetics is dependent on the capacity of oxygen delivery to the active muscle as well as oxygen utilization in the exercising muscle (Xu and Rhodes, 1999). VO_2_ kinetics are impaired in patients with HF (Poole et al., 2012) which is likely due to a reduced cardiac output, greater metabolic production during exercise, greater sympathetic activity, ventilatory work and recruitment of glycolytic fibers (Xu and Rhodes, 1999). When exercise in HF is above lactate threshold during dynamic exercise, which is often at low VO_2_ values, VO_2_ cost becomes greater, which is likely attributed to a combination of fatigue-related processes necessitating additional fiber recruitment and metabolic processes occurring within already recruited fibers (Poole et al., 2012; Jones and Poole, 2013). Consequently, slowed VO_2_ kinetics, although not yet been demonstrated during single limb fatiguing contractions, is likely contributing to greater fatigability in patients with HF.

During fatiguing contractions where force is maintained at > 60% of maximum, severe restriction of blood flow can occur (Sjøgaard et al., 1988). Importantly, it was demonstrated that during a maximal fatiguing contraction to 60% of the maximal voluntary contraction of the dorsiflexor muscles, patients with HF demonstrated accelerated fatigability (Minotti et al., 1992b), indicating that the greater fatigability was likely independent of blood flow. Further, when the muscle is made to be ischemic during dynamic knee extensor contractions, greater fatigability in patients with HF is observed (Minotti et al., 1991). Although not directly measured during a fatiguing contraction, this would collectively suggest that a lack of blood flow to the larger muscles likely contributes to greater fatigability (Figure 4), but also likely depends on the severity of HF (i.e., circulatory function).

#### Fatigability, Perceived Fatigue and Exercise Tolerance in HF: Are They Related?

How skeletal muscle fatigability, often considered performance fatigability, relates to symptoms of fatigue and exercise intolerance in patients with HF is an important question, yet challenging to answer. Studies that measured fatigability, did not report symptoms of fatigue during the fatiguing tasks. However, a number of these studies also conducted a peak oxygen consumption test (VO_2peak_) and perceived leg fatigue is measured during VO_2peak_ testing. Although the measurements of fatigability during single-limb tasks and whole-body VO_2peak_ testing are different, some mechanisms contributing to both may be similar (Longhurst et al., 1976; Wilson, 1995; Amann et al., 2014).

The importance of skeletal muscle fatigability to exercise intolerance is highlighted by earlier studies that demonstrated strong correlations between fatigability during dynamic isokinetic (90° and 180°/s velocities) contractions and VO_2peak_ (Minotti et al., 1991). Particularly at 90°/s, the reduction in strength was correlated with VO_2peak_ (*r* = 0.90, *r* < 0.001), such that those patients who had a greater reduction in strength after a dynamic fatiguing intervention also had the lowest VO_2peak_. Massie et al. (1996) observed a comparable finding with a similar fatiguing protocol (*r* = 0.57, *p* = 0.03) (Massie et al., 1996). This is particularly significant in view of the lack of correlation between VO_2peak_ and any measure of cardiac function. As of importance, both the fatigue index and VO_2peak_ correlated with the average fast twitch fiber area (Massie et al., 1996). Further, during VO_2peak_ testing, perceptions of leg fatigue was the greatest limiting factor reported by HF patients and was related to lower leg blood flow, greater oxygen extraction and greater muscle metabolism in HF patients (Wilson et al., 1984b, 1985). Collectively, findings from these studies suggest two important concepts: (1) skeletal muscle fatigability is closely associated to exercise capacity, a major measure of exercise intolerance in patients with HF and (2) some of the mechanisms (changes in fiber type distribution, muscle metabolism and blood perfusion to the muscle) that likely cause skeletal muscle fatigability are also major contributors to exercise intolerance in HF.

## Conclusion

Heart failure patients experience greater fatigability of the skeletal muscles, particularly in the lower extremity. Mechanisms contributing to the greater fatigability in patients with HF are likely due to alterations in skeletal muscle metabolism, resulting in greater glycolytic capacity and reduced oxidative capacity of the muscle and reduced blood perfusion to the muscle. A schematic of potential major contributors to performance fatigability during single-limb skeletal muscle contractions in HF is detailed in Figure 4. The abundance of work in fatigability in HF patients is from several decades ago and the majority of the mechanisms that may play a role have not been directly measured during fatiguing contractions in HF. As such, there are significant knowledge gaps in factors that contribute to fatigability in patients with HF, particularly the extent to which central or neural fatigability may play a role. Further, as some of the skeletal muscle changes have been observed in HFpEF, little is known in regards to mechanisms of fatigability in both HFrEF and HFpEF patients.

## Author Contributions

MK-R drafted, revised and approved the manuscript and agree to be accountable for all aspects of the work. ML revised and approved the manuscript and agree to be accountable for all aspects of the work. BJ revised and approved manuscript and agree to be accountable for all aspects of the work.

## Conflict of Interest Statement

The authors declare that the research was conducted in the absence of any commercial or financial relationships that could be construed as a potential conflict of interest.
